# Dynamic co-expression modular network analysis in nonalcoholic fatty liver disease

**DOI:** 10.1186/s41065-021-00196-8

**Published:** 2021-08-21

**Authors:** Jing Zheng, Huizhong Wu, Zhiying Zhang, Songqiang Yao

**Affiliations:** 1 Department of Pharmacy, Zhejiang Medical & Health Group Hangzhou Hospital, No.1 Banshan Road, Kangjian nong, Hangzhou, 310022 China; 2 Department of Pharmacy, Zhejiang Quhua Hospital, Quzhou, 324002 China; 3 Department of Pharmacy, Hangzhou Jianggan District People’s Hospital, Hangzhou, 310016 China

**Keywords:** Fatty liver, Obesity, Gene regulatory network

## Abstract

**Background:**

Nonalcoholic fatty liver disease (NAFLD) is the most common chronic liver disease affecting people’s health worldwide. Exploring the potential biomarkers and dynamic networks during NAFLD progression is urgently important.

**Material and methods:**

Differentially expressed genes (DEGs) in obesity, NAFL and NASH were screened from GSE126848 and GSE130970, respectively. Gene set enrichment analysis of DEGs was conducted to reveal the Gene Ontology (GO) biological process in each period. Dynamic molecular networks were constructed by DyNet to illustrate the common and distinct progression of health- or obesity-derived NAFLD. The dynamic co-expression modular analysis was carried out by CEMiTool to elucidate the key modulators, networks, and enriched pathways during NAFLD.

**Results:**

A total of 453 DEGs were filtered from obesity, NAFL and NASH periods. Function annotation showed that health-NAFLD sequence was mainly associated with dysfunction of metabolic syndrome pathways, while obesity-NAFLD sequence exhibited dysregulation of Cell cycle and Cellular senescence pathways. Nine nodes including COL3A1, CXCL9, CYCS, CXCL10, THY1, COL1A2, SAA1, CDKN1A, and JUN in the dynamic networks were commonly identified in health- and obesity-derived NAFLD. Moreover, CYCS, whose role is unknown in NAFLD, possessed the highest correlation with NAFLD activity score, lobular inflammation grade, and the cytological ballooning grade. Dynamic co-expression modular analysis showed that module 4 was activated in NAFL and NASH, while module 3 was inhibited at NAFLD stages. Module 3 was negatively correlated with CXCL10, and module 4 was positively correlated with COL1A2 and THY1.

**Conclusion:**

Dynamic network analysis and dynamic gene co-expression modular analysis identified a nine-gene signature as the potential key regulator in NAFLD progression, which provided comprehensive regulatory mechanisms underlying NAFLD progression.

**Supplementary Information:**

The online version contains supplementary material available at 10.1186/s41065-021-00196-8.

## Background

Nonalcoholic fatty liver disease (NAFLD) is regarded as one of the most common chronic liver diseases. NAFLD is associated with obesity, and ranges from simple hepatic steatosis (nonalcoholic fatty liver [NAFL]) to nonalcoholic steatohepatitis (NASH). Some patients with NAFLD eventually developed cirrhosis, fibrosis, or hepatocellular carcinoma [1]. Since the liver is required for the glucose metabolism process and energy homeostasis, NAFLD also acts as a high-risk factor for metabolic diseases such as type 2 diabetes. A recent study confirmed that NAFLD is closely related to high mortality, especially liver disease-specific and diabetes-specific deaths [2]. Due to the high prevalence, high mortality, and serious complications, exploring the potential biomarkers and elucidating the dynamic networks involved in NAFLD are becoming urgently important.

NAFLD is mainly caused by obesity. Lifestyle intervention such as weight loss with diet or surgery is a primary therapy for NAFLD [3]. Liver transplantation has been proven to be an efficient method to treat NAFLD especially at the end stage [4], but the shortage of the donor’s liver and surgical complications limit the therapy. The treatment of NAFLD is to prevent NAFL from developing to NASH, which is highly associated with liver cirrhosis, fibrosis or serious hepatic complications. However, there is no specific drug approved for NAFLD until now. Moreover, NAFLD is increasingly being identified in non-obese patients. The common and distinct mechanisms involved in the non-obesity and obesity-derived NAFLD has not been fully elucidated. Expression profiling by high throughput sequencing has been a powerful tool to reveal key factors and pathways in NAFLD liver, and some studies have focused on the different stages of NAFLD [5, 6]. However, the dynamic alterations in key genes and co-expression networks were not fully elucidated during NAFLD progression. Searching key regulators, functional networks, and pathways in obesity, NAFL and NASH would be helpful to prevent and delay the development of NAFLD.

In this work, we focused on the dynamic gene landscape and pathways during NAFLD progression. Consistently changed genes were screened from GSE126848 [5] and GSE130970 [7]. Dynamic molecular networks and dynamic co-expression modular analysis were constructed to illustrate the key modulators, networks, and enriched pathways during NAFLD. The results would offer new clinical biomarkers for different stages of NAFLD, and provide potential drug targets for NAFLD treatment.

## Material and methods

### Microarray data

The flow diagram of this study was illustrated in supplementary figure 1. The search strategy is “(NAFLD) OR (nonalcoholic fatty liver disease)” in GEO (http://www.ncbi.nlm.nih.gov/geo/). The criteria are: 1. The dataset is an RNA sequencing dataset; 2. The dataset has more than 10 samples; 3. The dataset contains healthy, NAFL and NASH subjects. Finally, two datasets GSE126848 and GSE130970 meet the criteria. The RNA sequencing dataset GSE126848 was based on platform GPL18573 [5]. The liver biopsies comprised 14 healthy donors, 12 obese, 15 NAFL and 16 NASH patients. The dataset GSE130970 was based on platform GPL16791 [7]. According to the NIDDK NASH CRN criteria, 26 samples with the NAFLD activity score (NAS) ≥ 5 were regarded as NASH, 10 samples with steatosis and NAS < 3 were considered as NAFL, 4 samples with NAS = 0 was chosen as normal tissues. Due to the limited histological information, the liver histology of the rest 36 samples could not be defined and these samples were not enrolled in our analysis.

### Data processing and identification of DEGs

Basing on R (version 3.5.1), rtracklayer and SummarizedExperiment packages were applied to annotate the raw data using the human genome reference GRCh38 [8]. The edgeR package was applied to conduct the background correction, normalization and log2-counts-per-million transformation. Empirical Bayes method depending on the limma package was performed to identify DEGs of GSE126848 and GSE130970. The thresholds for DEGs filtration were set as |log2 fold change (FC)|≥ 1 and *P* value < 0.05. Since only GSE126848 contained the obese samples, the DEGs in obesity were filtered from GSE126848. Common DEGs in NAFL and NASH were screened by the intersection of GSE126848 and GSE130970. GSE126848 contained all of the three disease types, therefore, the expression profiles of these DEGs at different stages were then extracted from GSE126848 for the dynamic expression pattern and gene set enrichment analysis.

### Gene set enrichment analysis of DEGs

Gene set enrichment analysis (GSEA) of the DEGs was conducted using gseaGO function within the R package clusterProfiler [9]. The permutation number was set as 1500. The org.Hs.eg.db package was applied to map gene identifiers. Ridgeline plots were performed using ggridges and ggplot2 packages. Gene set with a *P* value < 0.05 was considered to be significant.

### Analysis of DEGs expression pattern during NAFLD progression

The union of DEGs in obesity, NAFL and NASH was input and normalized by Short Time-series Expression Miner (STEM) to explain the NAFLD progression. STEM is a software designed for clustering, comparing, and displaying gene expression data from time-course experiments [10]. The maximum number of expression models was set as 60, and *P* values were adjusted using FDR. The other parameters were set as default. Clusters with colors mean statistically significant numbers of genes enriched.

### Analysis of dynamic molecular interaction networks during NAFLD progression

To analysis the key genes involved in the NAFLD progression, dynamic molecular interaction networks were constructed using DyNet (1.0.0) application in Cytoscape (3.6.1). DEGs were input into the STRING database to construct the protein–protein interaction (PPI) network, and DyNet [11] was applied to analyze the most ‘rewired’ nodes across dynamic network states. To analyze the heterogeneous mechanism of NAFLD, the rewired nodes from obese and non-obese NALFD progression sequences were intersected and subsequently enriched by KEGG within the R package clusterProfiler [9]. Node degree variance was calculated by DyNet and was displayed as Dn-Score.

### Dynamic co-expression modular analysis

The CEMiTool is a comprehensive R package that allowed the users to identify co-expressed gene modules, hubs, and determine significant module functions [12]. The R packages WGCNA and CEMiTool were combined to calculate the correlation between key nodes identified above and gene co-expression modules. A *P* < 0.05 was defined as significant correlation. Then, files including GO gene sets (C5) from MSigDB 7.0 and human protein–protein interaction (PPI) from the mentha database were regarded as enrichment and PPI background. The association of module activity was determined using fgsea package. The biological functions related to modules were annotated using the over representation analysis (ORA).

## Results

### Function annotation of DEGs in obese, NAFL and NASH patients

**Fig. 1:**
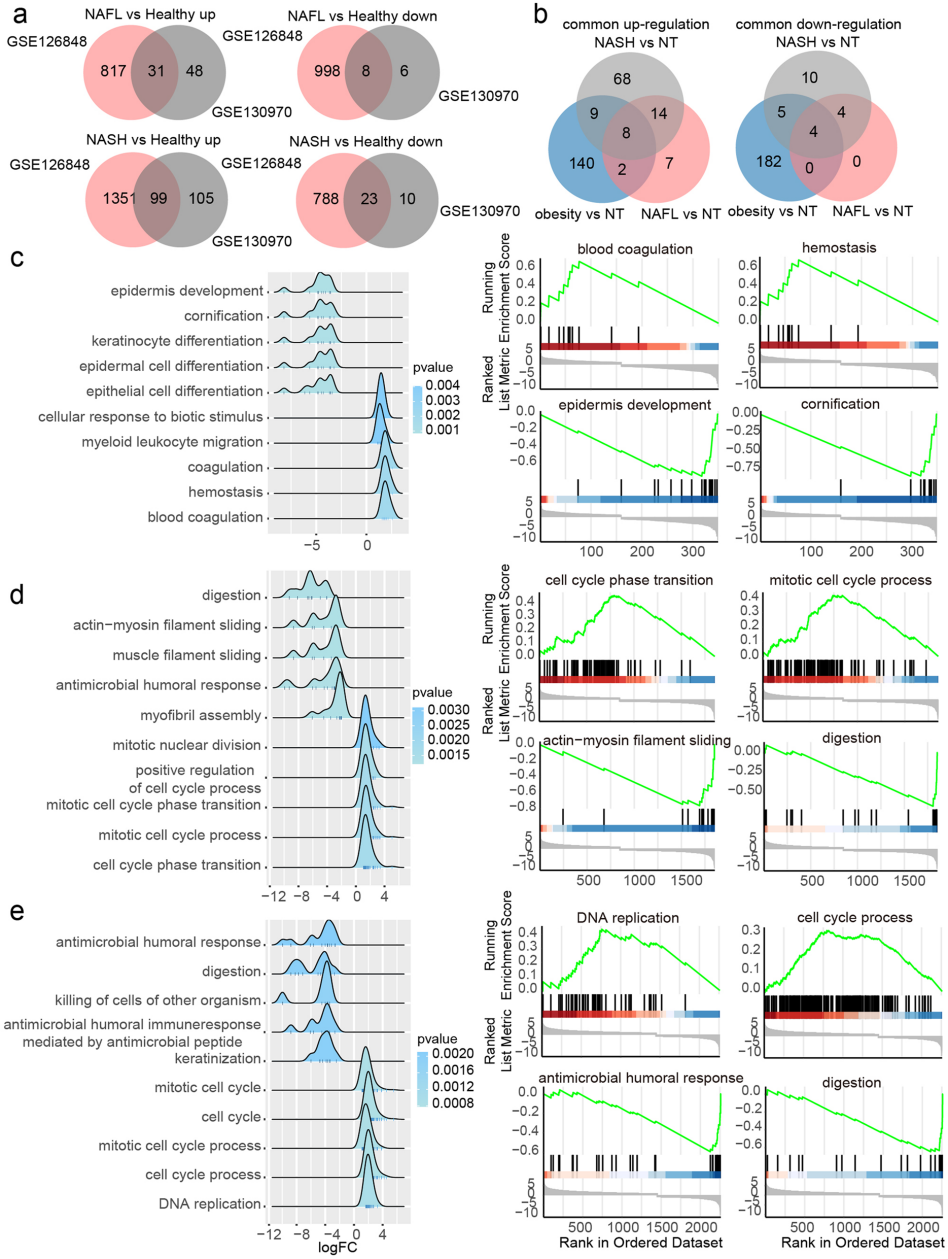
Function annotation of DEGs in obese, NAFL and NASH patients by GSEA. **A** Consistently changed DEGs between health, NAFL and NASH in GSE126848 and GSE130970. **B** DEGs in obesity NAFL and NASH. **C**-**E** The top 5 significant upregulated and downregulated gene sets based on normalized enrichment score (NES) were presented as ridgeline plots (**C** obese; **D** NAFL; **E** NASH). The top 2 upregulated and downregulated gene sets in disease groups (**C** obesity; **D** NAFL; **E** NASH) were ranked by NES on the right

GEO datasets GSE130970 and GSE126848 were enrolled in our study, and the clinicopathological features of patients in two datasets were presented in Table 1. DEGs in NAFL and NASH were firstly screened by the intersection of GSE126848 and GSE130970, respectively. As a result, 31 upregulated DEGs and 8 downregulated DEGs were filtered from NAFL samples, while 99 upregulated DEGs and 23 downregulated DEGs were screened from NASH subjects (Fig. 1A). Moreover, 159 upregulated DEGs and 191 downregulated DEGs were found in obese patients. The Venn diagram showed that 8 upregulated DEGs (including TP53I3, FNDC5, INHBE, SERPINE1, PRKCE, ACSL4, IP6K3 and CXCL10), and 4 downregulated DEGs (including B3GAT1, P4HA1, IGFBP2 and GPR88) were shared by all three groups (Fig. 1B).

**Table 1:** Clinicopathological features of patients in different datasets

Variables	**GSE130970**			**GSE126848**			
	NT	NAFL	NASH	NT	Obesity	NAFL	NASH
Age (years)							
Mean	53	50.5	53.9	/	/	/	/
Gender							
Male	1	5	9	14	12	9	12
Female	3	5	17	0	0	6	4
Lobular inflammation grade							
0	4	3	0	/	/	/	/
1	0	7	18	/	/	/	/
2	0	0	8	/	/	/	/
Cytological ballooning grade							
0	4	10	1	/	/	/	/
1	0	0	7	/	/	/	/
2	0	0	18	/	/	/	/
Steatosis grade							
0	4	0	0	/	/	/	/
1	0	9	2	/	/	/	/
2	0	1	13	/	/	/	/
3	0	0	11	/	/	/	/
Fibrosis stage							
0	4	7	1	/	/	/	/
1	0	3	12	/	/	/	/
2	0	0	4	/	/	/	/
3	0	0	8	/	/	/	/
4	0	0	1	/	/	/	/
NAFLD activity score							
NAS = 0	4	0	0	/	/	/	/
0 < NAS < 3	0	10	0	/	/	/	/
NAS ≥ 5	0	0	26	/	/	/	/

In order to elucidate the functional gene sets involved in the process of obesity, NAFL and NASH, we conducted the GO biological process enrichment using DEGs from each period, respectively. In the obesity group, the top 2 upregulated gene sets ranked by normalized enrichment score (NES) were blood coagulation, hemostasis, and the top 2 downregulated gene sets were epidermis development, cornification (Fig. 1C). In the NAFL group, the top 2 upregulated gene sets were cell cycle phase transition, mitotic cell cycle process, and the top 2 downregulated gene sets were actin-myosin filament sliding, digestion (Fig. 1D). For DEGs in NASH, the top 2 upregulated gene sets were DNA replication, cell cycle process, and the top 2 downregulated gene sets were antimicrobial humoral response, digestion (Fig. 1E).

### Dynamic landscape of DEGs during NAFLD progression

**Fig. 2:**
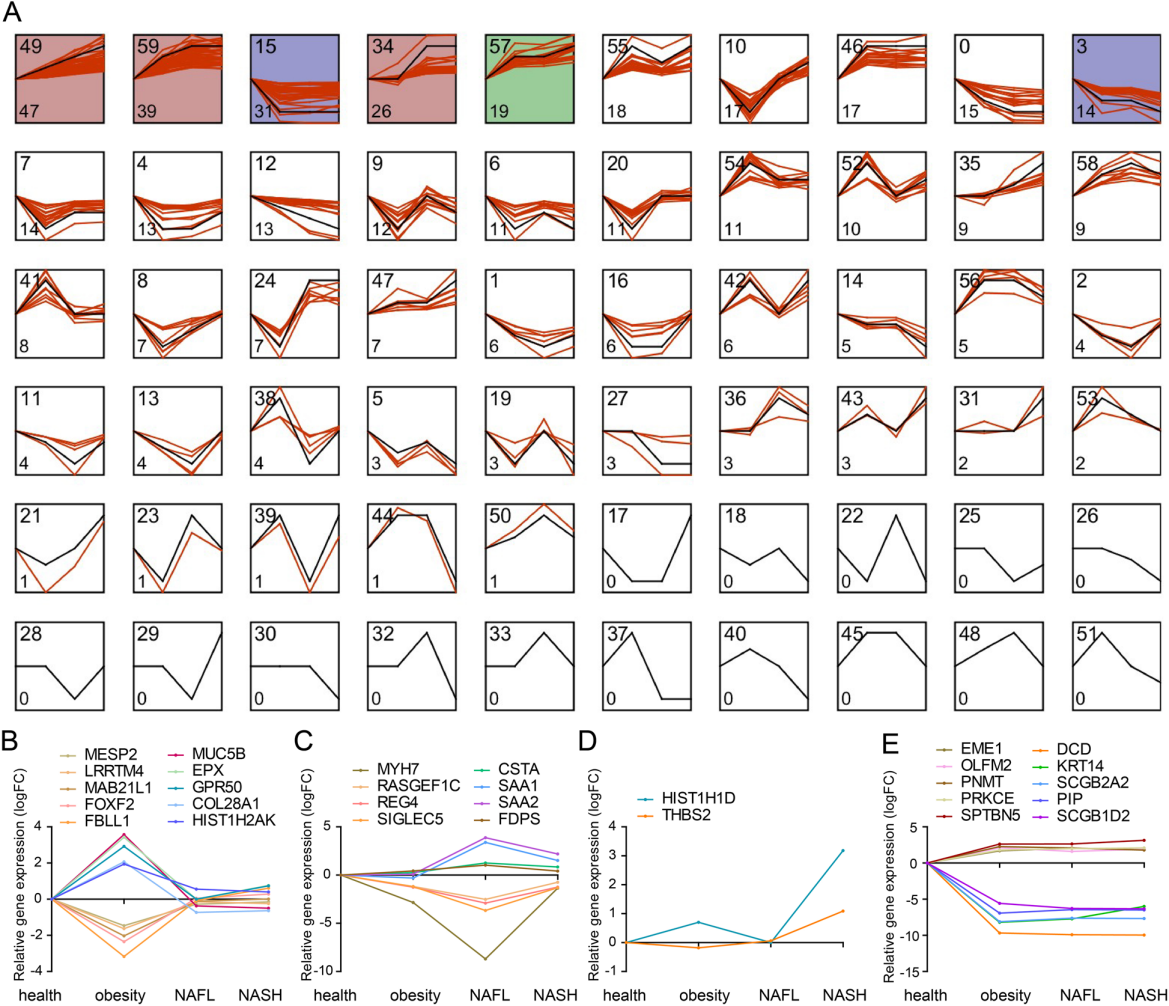
Dynamic expression patterns of DEGs during NAFLD progression. **A** Dynamic expression profiles of DEGs. Each box represents a temporal expression model profile. The black line indicates the model expression trend. The red lines represent the individual gene expression at different periods. The profile ID is shown in the top left corner, and the number of gene in the profile is shown in the bottom left corner. The colored profiles indicate that a statistically significant number of genes was assigned to the profile. **B**–**E** Classified gene expression trends in the 4 meaningful trends were present in different stages. The top five DEGs ranked by expression fold changes in each profile were presented in the line charts

To further explore the markers during NAFLD progress, dynamic profiles of all DEGs in Fig. 1B were analyzed. Altogether 60 expression trends were determined in our study. Here we summarized four featured expression trends among these profiles, and the top five DEGs ranked by expression fold changes in each profile were presented in the line charts. The first trend was that the DEGs specifically changed in obesity group, including profile 41 and profile 20 (Fig. 2B). The second trend was that the DEGs particularly changed in NAFL group. Profile 36 and profile 50 were upregulated, and profile 11 was downregulated in NAFL compared with other stages (Fig. 2C). The third trend was DEGs only changed in NASH group. HIST1H1D and THBS2 in profile 31 was in line with this trend (Fig. 2D). The fourth trend was that DEGs changed in all three stages. We found that DEGs in profile 46, 49,59, 57, 55, 54, 58, 47, 56 were upregulated, and profile 15,0, 3, 12, 6, 1, 14 were downregulated (Supplementary table 1). For example, EME1, OLFM2, PNMT, PRKCE, SPTBN5 in profile 46 were upregulated, and DCD, KRT14, SCGB2A2, PIP, SCGB1D2 in profile 15 were downregulated in all three periods (Fig. 2E).

### Dynamic network analysis in health- and obesity-NAFL-NASH sequences

**Fig. 3:**
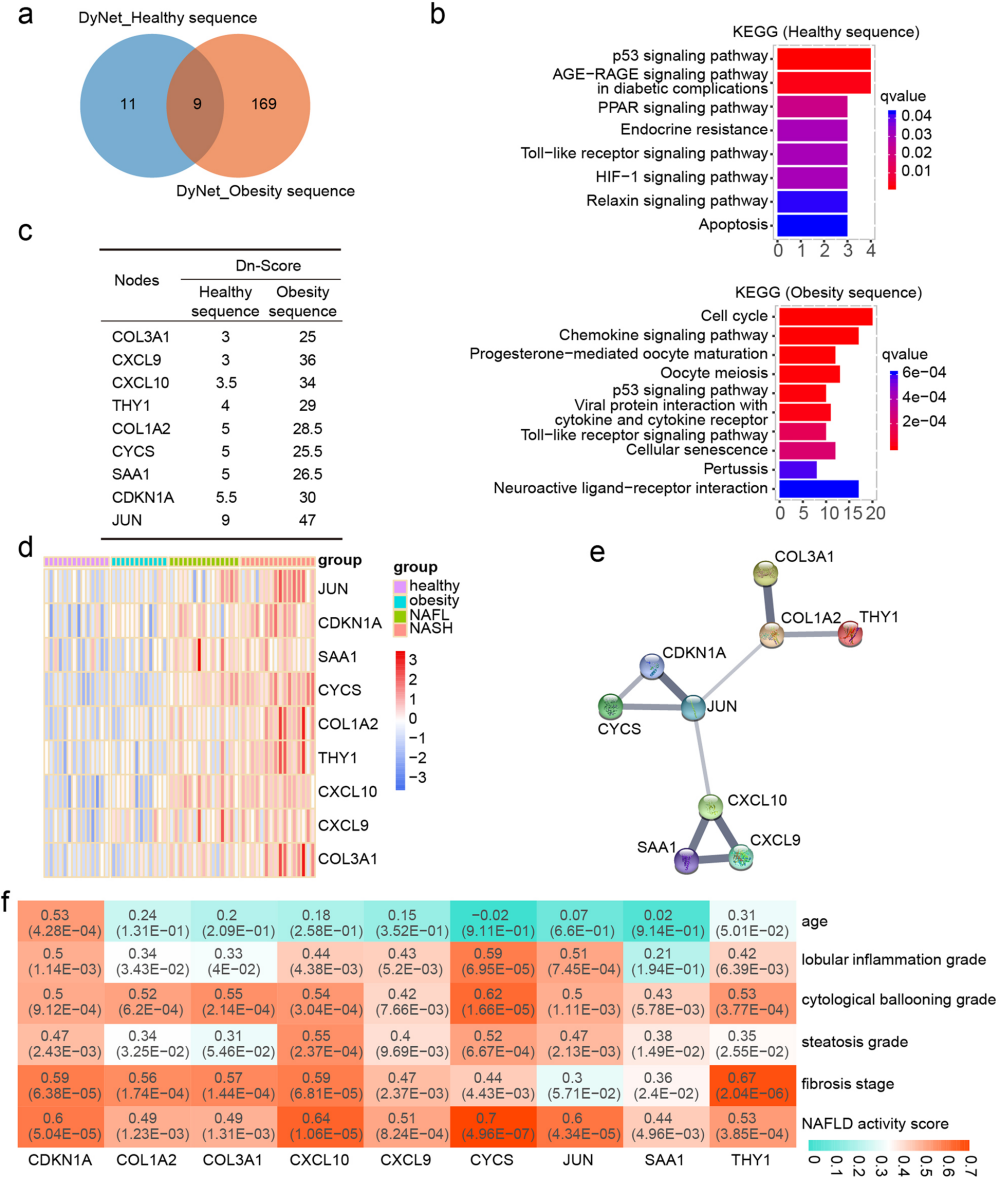
Dynamic molecular networks along the NAFLD progression sequence. **A** The node genes involved in dynamic networks of obesity and non-obesity derived NAFLD. Dynamic networks were created and Dn-Scores of node genes were calculated by DyNet. **B** Pathway enrichment of node genes in healthy- or obesity-NAFLD sequences, respectively. **C** The Dn-Score of common nodes shared by healthy- and obesity-NAFLD sequences. **D** Expression heatmap of nine common nodes at different NAFLD stages. **E** The protein–protein interactions of nine common nodes were retrieved by the STRING database. **F** Pearson correlation analysis of the nine nodes and clinical parameters of NAFLD

To explore the common and distinct mechanisms of healthy or obese subjects-derived NAFLD, dynamic networks were constructed by Dynet in the Cytoscape software. As a result, 20 key nodes in the health-NAFL-NASH sequence and 178 key nodes in the obesity-NAFL-NASH sequence were identified by DyNet (Fig. 3A). Distinct pathways of these rewired nodes in different sequences were analyzed by KEGG (Fig. 3B). Only eight pathways including p53 signaling pathway, AGE − RAGE signaling pathway in diabetic complications, PPAR signaling pathway, Endocrine resistance, Toll − like receptor signaling pathway, HIF-1 signaling pathway, Relaxin signaling pathway, and Apoptosis were significantly enriched in the health-NAFL-NASH sequence. The top 10 KEGG pathways enriched in obesity-NAFL-NASH sequence included cell cycle, Chemokine signaling pathway, Progesterone-mediated oocyte maturation, Oocyte meiosis, p53 signaling pathway, Viral protein interaction with cytokine and cytokine receptor, Toll − like receptor signaling pathway, Cellular senescence, Pertussis and Neuroactive ligand − receptor interaction (Fig. 3B). Nine rewired nodes COL3A1, CXCL9, CYCS, CXCL10, THY1, COL1A2, SAA1, CDKN1A, and JUN were commonly included in both health- and obesity-NAFL-NASH sequences (Fig. 3C). The expression levels of these nine node genes were all up-regulated during NAFLD progression derived from obese or non-obese individuals (Fig. 3D), suggesting these nine genes may play fundamental roles in NAFLD development. The PPI network of these nine key nodes was displayed in Fig. 3E.

We next analyzed the correlation between these nine genes and the clinical parameters (Fig. 3F). We demonstrated that all of the nine genes were positively correlated with the NAFLD activity score, and CYCS has the highest correlation (*r* = 0.7, *P* = 4.96 × 10^–7^). CDKN1A (*r* = 0.53, *p* = 4.28 × 10^–4^) was the most relevant gene with age. Moreover, CYCS was the most relevant gene with the lobular inflammation grade (*r* = 0.59, *P* = 6.95 × 10^–5^) and the cytological ballooning grade (*r* = 0.62, *P* = 1.66 × 10^–5^). CXCL10 (*r* = 0.55, *P* = 2.37 × 10^–4^) was the most relevant gene with the steatosis grade, and THY1 (*r* = 0.67, *P* = 2.04 × 10^–6^) was the most relevant gene with the fibrosis stage.

### Identification of dynamic co-expression module during NAFLD progression

**Fig. 4:**
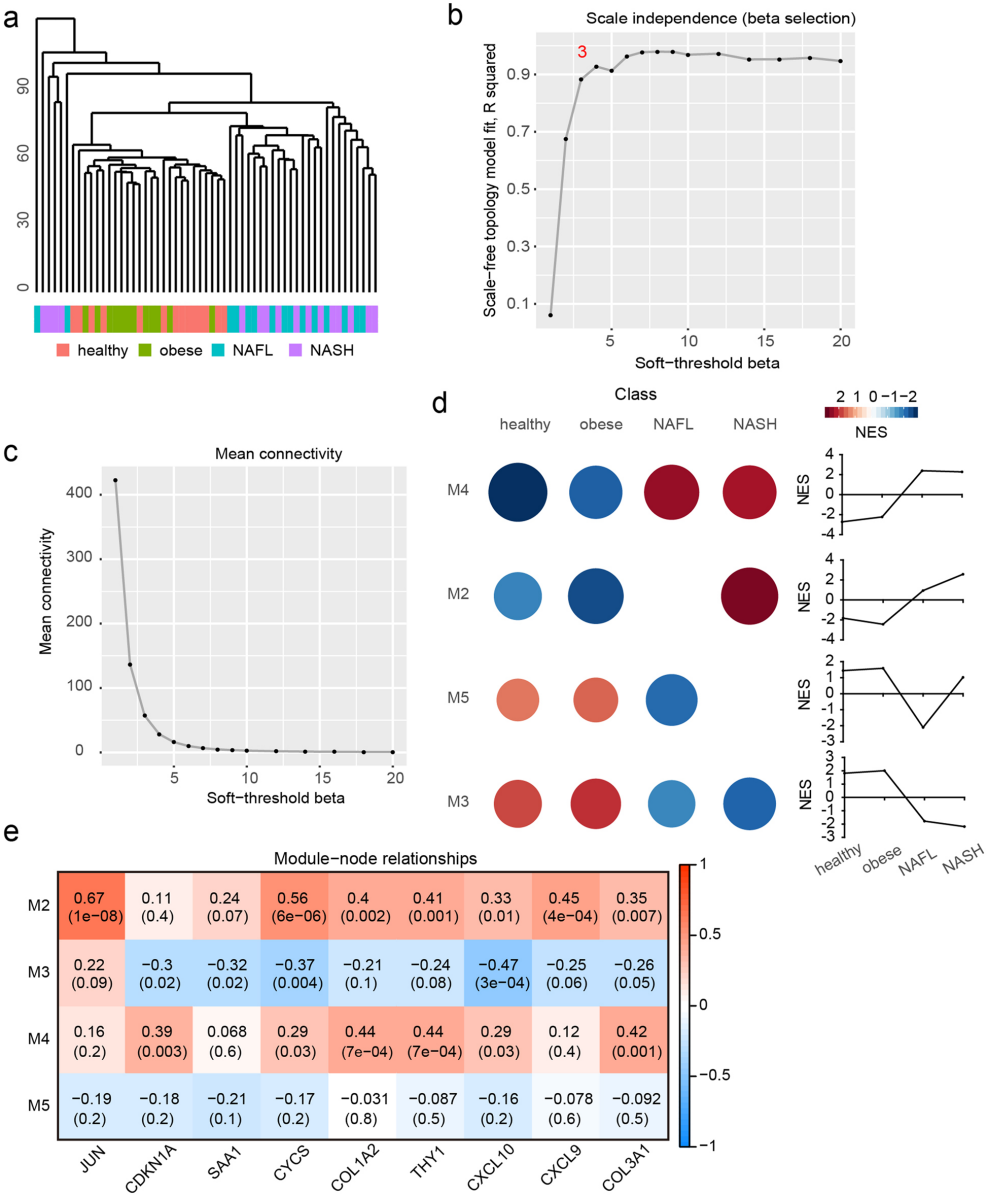
Dynamic co-expression module identification during NAFLD progression. **A** Sample dendrogram and clinical trait heatmap. **B** Analysis of scale independence for various soft-thresholding powers. **C** Analysis of mean connectivity for various soft-thresholding powers. **D** Modules were enriched by co-expression network analysis. The activities of modules at different stage during NAFLD progression were calculated by NES from GSEA, and the modules enriched in each stage with adjust *P* value < 0.05 were shown in heatmap. **E** Module-node relationships were assessed by WGCNA. Nine common node genes identified above were selected to evaluate the correlation with Modules

To investigate the dynamic co-expression networks during NAFLD progression, CEMiTool was carried out to disclose highly correlated gene modules. The sample dendrogram and clinical trait heatmap were displayed in Fig. 4A. A power of β = 3 was selected by scale independence and mean connectivity (Fig. 4B and C). Five modules were enriched by co-expression network analysis. Module 4 (M4) was inhibited in health and obesity groups, and activated in NAFL and NASH. M2 was inhibited in health and obesity groups, and only activated in NASH. M5 was activated in health and obesity groups, and only inhibited in NAFL. M3 was activated in health and obesity groups, and inhibited in NAFL and NASH (Fig. 4D). The relationships between these modules and nine fundamental nodes from dynamic networks were analyzed by WGCNA (Fig. 4E). JUN, CYCS, COL1A2, THY1, CXCL10, CXCL9 and COL3A1 were positively correlated with M2. CDKN1A, SAA1, CYCS, CXCL10 were negatively correlated with M3. CDKN1A, CYCS, COL1A2, THY1, CXCL10, and COL3A1 were positively correlated with M4. None of the nine rewired nodes were significantly correlated with M5.

### Gene interaction networks of dynamic co-expression modules

**Fig. 5:**
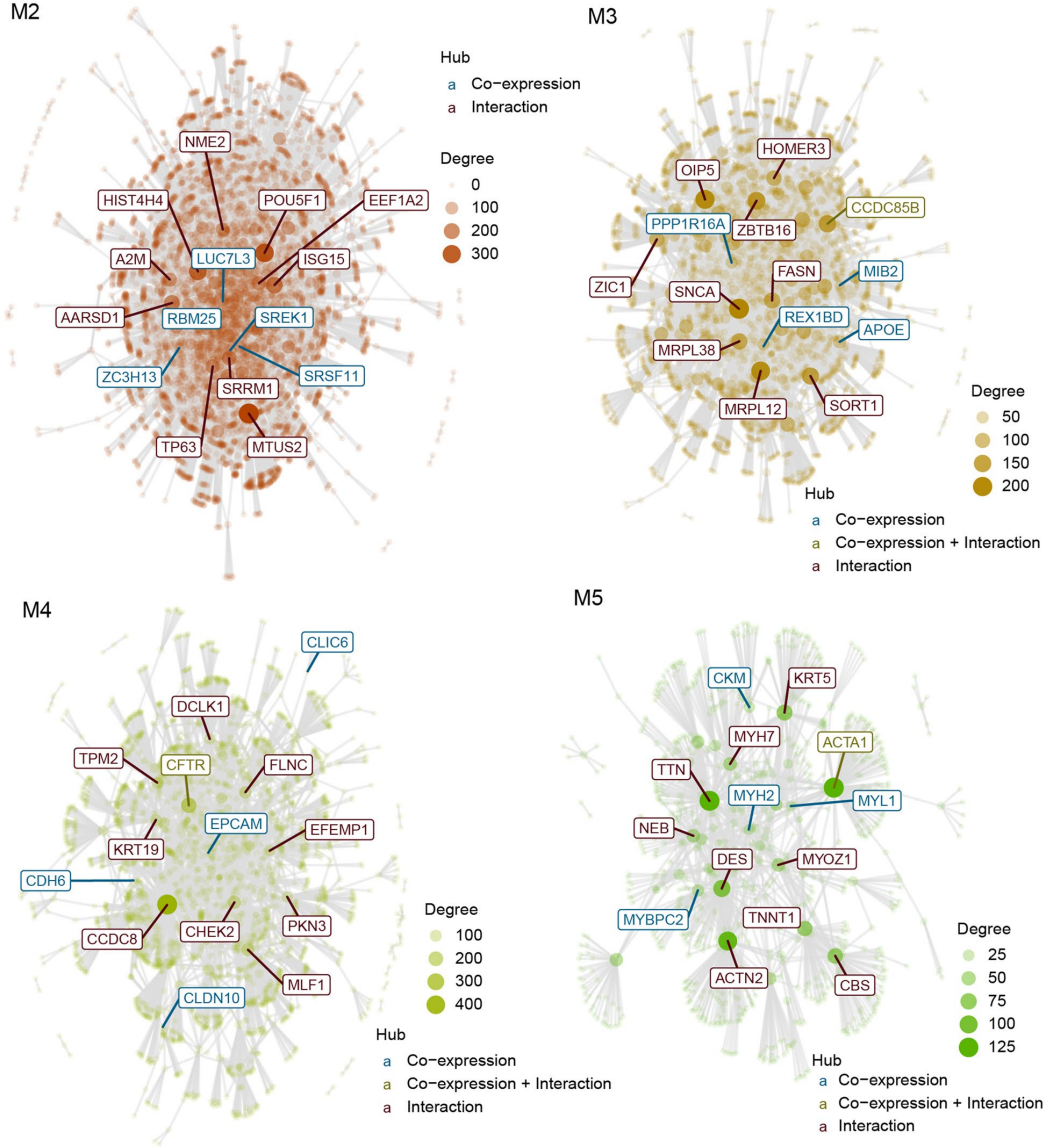
Gene interaction networks of dynamic co-expression modular analysis. The size and color of the nodes were proportional to their degrees. The most connected hubs in each module were labelled in the networks. The co-expression hubs which originally presented in the CEMiTool module were colored blue, while the hubs which inserted from the interaction file were colored red. The hub which acted as co-expression hub and interaction hub was colored green

Then, we analyzed the gene networks of the dynamic modules (Fig. 5). SREK1, SRSF11, ZC3H13, RBM25, LUC7L3 were co-expression hubs of M2. NME2, HIST4H4, POU5F1, EEF1A2, A2M, ISG15, AARSD1, SRRM1, TP63, and MTUS2 were interaction hubs of M2. MIB2, REX1BD, PPP1R16A, APOE, CCDC85B were co-expression hubs of M3. HOMER3, OIP5, ZBTB16, ZIC1, SNCA, FASN, CCDC85B, MRPL38, MRPL12, and SORT1 were interaction hubs of M3. CLDN10, EPCAM, CFTR, CDH6, CLIC6 were co-expression hubs of M4. DCLK1, TPM2, FLNC, KRT19, EFEMP1, CCDC8, CHEK2, PKN3, CFTR and MLF1 were interaction hubs of M4. MYL1, ACTA1, CKM, MYH2, MYBPC2 were co-expression hubs of M5. KRT5, MYH7, TTN, NEB, DES, MYL1, MYOZ1, TNNT1, CBS and ACTN2 were interaction hubs of M5.

### Functional pathway annotation of modules by ORA

**Fig. 6:**
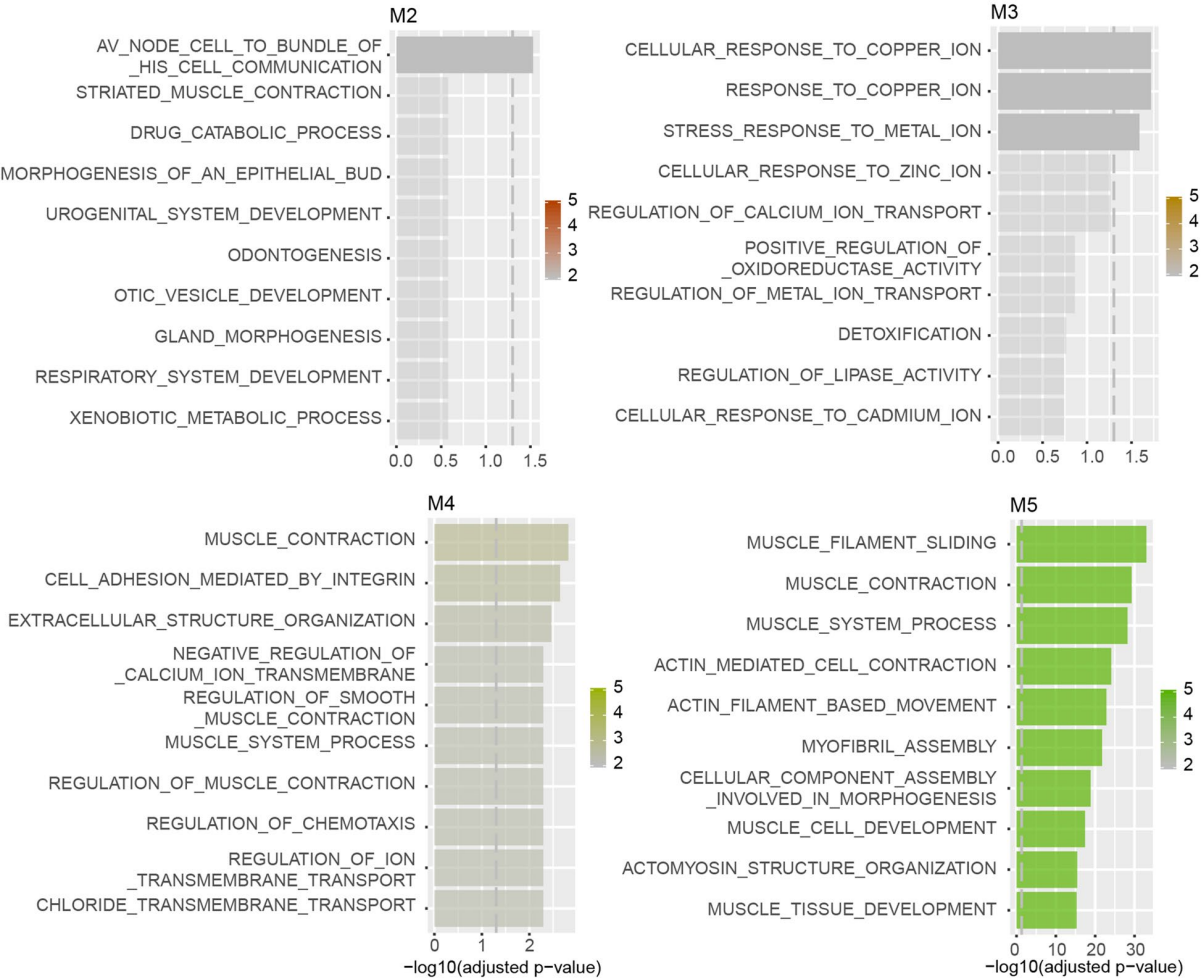
Functional pathway annotation of modules by ORA. ORA was conducted to search the Gene Ontology (GO) biological process in four modules generated by CEMiTool. Top ten pathways rank by adjust *p* value enriched in each module were listed in Bar graphs. The bar of pathway which cross the dashed grey line indicated a significant pathway with adjust *P* value < 0.05

ORA was conducted to search the GO biological process in modules (Fig. 6). Av node cell to bundle of his cell communication was enriched in M2. Response to copper ion, cellular response to copper ion, and stress response to metal ion were enriched in M3. Muscle contraction, cell adhesion mediated by integrin, extracellular structure organization, negative regulation of calcium ion transmembrane transport, regulation of chemotaxis, regulation of muscle contraction, muscle system process, regulation of smooth muscle contraction, and negative regulation of ion transmembrane transport were enriched in M4. Muscle filament sliding, muscle contraction, muscle system process, actin mediated cell contraction, actin filament based movement, myofibril assembly, cellular component assembly involved in morphogenesis, muscle cell development, actomyosin structure organization, and muscle tissue development were enriched in M5.

## Discussion

NAFLD represents a spectrum of liver disorders including NAFL and NASH, while NASH is tightly associated with the end stage of liver disease [13]. Obesity is one of the main causes of NAFLD. Thus, it is urgent to prevent the progressions from obesity to NAFL and from NAFL to NASH. Here, we attempted to explore the key molecular pathways and dynamic co-expression networks along the NAFLD progression course.

Functional pathway annotation of DEGs showed that cell cycle related pathways were upregulated in NAFL and NASH. It is consistent with previous research that canagliflozin could attenuate the development of NASH partly by the induction of cell cycle arrest [14]. Our data indicated that cell cycle related pathways also participated in NAFL. Aravinthan A found the permanent cell cycle arrest in NAFLD [15], which indicated an opposite role in NAFLD. Therefore, the roles of cell cycle in NAFLD need further exploration.

To investigate the gene alteration trends from obesity to NASH, the dynamic profiles of DEGs were conducted. In the current study, we screened several expression models that specifically changed at particular disease stages, which could be novel markers to identifying obesity, NAFL and NASH, respectively. SAA1 and SAA2 are serum amyloid A family members, and only upregulated in NAFL. They are highly expressed in response to liver inflammation [16], and play important roles in lipid metabolism [17]. Moreover, SAA1 was one of the 9 rewired nodes in health- and obesity-NAFL-NASH sequences. SAA1 acted as a hub gene of PPI network constructed by DEGs from NAFLD dataset GSE106737 and GSE83452 [18]. Increased hepatocyte SAA1 aggravated liver inflammation in NAFLD [19]. Our findings suggested that SAA1 and SAA2 may play key roles in early NAFL stage. THBS2 only upregulated in NASH. It has been proved that the gene expression of THBS2 increased in fatty liver of NAFLD [20]. THBS2 increased during diet-induced mice hepatic fibrosis progression [21] and was up-regulated in the fibrosis stage 3–4 state of NAFLD patients [22]. We speculated that THBS2 might be related to the hepatic fibrosis in the late NASH period. Besides, we also found DEGs that were obviously changed in all periods. PRKCE is a member of the Protein kinase C family. Protein kinase C members, such as PKCδ, are closely related to insulin sensitivity and body glucose tolerance [23]. Activation of PRKCE links the NAFLD to hepatic insulin resistance [24, 25]. PRKCE has been proven to be a critical DEG in NAFLD, and the expression level and DNA methylation of PRKCE and IGFBP2 were altered in obesity-derived NAFLD [26]. Hepatic steatosis leads to hepatic insulin resistance by activating PRKCE [27]. These results were consistent with our data, PRKCE kept upregulating at obesity, NAFL, and NASH stages. We considered that the PRKCE regulated the lipid abundance, steatosis, insulin resistance, thereby modulated the pathogenetic process of NAFLD. These results indicated the important roles of these DEGs in obese and NAFLD development.

Although NAFLD is commonly associated with obesity, it is increasingly being identified in non-obese patients. Therefore, we further investigate the common and distinct mechanisms between the health-NAFL-NASH sequence and the obesity-NAFL-NASH sequence. The pathophysiology of non-obese NAFLD is still not clear [28]. It has been reported that metabolic syndrome promotes the progression to NASH in non-obese patients with NAFLD [29]. Our result indicated that AGE − RAGE signaling pathway in diabetic complications, PPAR signaling pathway, Endocrine resistance were enriched in healthy-NAFL-NASH sequence, which are all associated with metabolic syndrome. On the other hand, the obesity-NAFLD sequence was mainly enriched in cell cycle, chemokine signaling pathway, cellular senescence.

By combination analysis of dynamic networks and dynamic co-expression modules, we also identified nine genes that essential for the progression of NAFLD derived from obese and non-obese individuals. These genes mainly contained collagens, chemokines and oncogenes. CYCS has the highest correlation with NAFLD activity score, lobular inflammation grade and the cytological ballooning grade. CYCS functions as a central component of the electron transport chain in mitochondria. Although the role and effect of CYCS during NAFLD progression have not been explored, a recent study demonstrated that CYCS was associated with hepatic lipid metabolic misalignment [30]. These findings suggested that CYCS might be a novel regulator in promoting the progression of NAFLD. The PPI network showed that CYCS interacted with CDKN1A and JUN. JUN was most positively correlated with M2. JUN and EGR were reported to drive the reprogramming of the Kupffer cell to a scar-associated macrophage phenotype by changing the liver X receptor functions during diet-induced NASH [31]. We also demonstrated that M2 was only activated in NASH, and we surmised that this module may contribute to liver fibrosis to some extent. M3 was inhibited in NAFL and NASH, and was most negatively correlated with CXCL10. CXCL10 has been reported to inhibit autophagic protein degradation and the accumulation of ubiquitinated proteins, thereby promoted the development of steatohepatitis [32]. Blockade of CXCL10 protected against steatohepatitis development in mice, and CXCL10 levels were significantly higher in human NASH [33–35]. We found that CXCL10 upregulated in NAFL and NASH samples, and was indeed the most relevant gene with the steatosis grade. Our result demonstrated that M4 was activated in NAFL and NASH, and may relate to the development of NAFLD as well. M4 was most positively correlated with COL1A2 and THY1. It has been reported that in severe NAFLD patients the levels of pro-IL1β mRNA correlate with the expression of COL1A1 [36]. Similar to COL1A1, COL1A2 is also a fibrosis-related gene [37], however, the role of COL1A2 in NAFLD has not been fully elucidated. We found that THY1 was the most relevant gene with the fibrosis stage. THY1 encodes a cell surface glycoprotein and member of the immunoglobulin superfamily of proteins, while little is known about its effects on NAFLD.

Pathway enrichment showed that response to copper ion and cellular response to copper ion were enriched in M3. Copper has been proven to play a major role in NAFLD [38], and copper homeostasis could counteract the progression of NAFLD [39]. In the hub genes of M3, SNCA and APOE were reported to be associated with copper ion related pathways. SNCA interacts with copper in Parkinson’s disease [40]. APOE deletion has no effect on copper-induced oxidative stress in the mice brain [41], but APOE might participate in protecting the liver from copper-induced damage [42]. The relationships between hub genes and copper ion related pathways remained unclear in NAFLD. FASN was an interaction hub gene in M3. FASN catalyzed the synthesis of palmitate and long-chain saturated fatty acids [43]. FASN expression was correlated significantly with the degree of hepatic steatosis, but not with inflammation or ballooning of hepatocytes [44]. The result was similar to another M3 correlated gene CXCL10, which was also relevant to the steatosis grade. Therefore, we speculated that M3 may closely related to hepatic steatosis. Cell adhesion mediated by integrin and regulation of chemotaxis were enriched in M4. Loss of cell adhesion molecule-1 had beneficial effects in NASH development by reducing inflammation, and β7-integrin-deficiency results in increased steatohepatitis[45]. In the hub genes of M4, EPCAM and EFEMP1 were associated with NAFLD. EPCAM, the epithelial cell adhesion molecule was upregulated in NASH [46]. EFEMP1 was identified as a hub gene in NAFLD fibrosis [22].

## Conclusion

In summary, our study identified a nine-gene signature as the potential key regulator in NAFLD progression. The results provided potential pivotal genes and the clinical markers during NAFLD progression. Further experiments are still needed to explore the function of dynamic co-expression networks in NAFLD.

## Supplementary Information

**Additional file 1: Supplementary figure 1.** The flow diagram of dynamic network analysis.

**Additional file 2: Supplementary table 1.** DEGs in different profiles.

## Data Availability

The data are available from the corresponding author on reasonable request.

## Abbreviations

NAFLD: Nonalcoholic fatty liver disease
DEGs: Differentially expressed genes
NASH: Nonalcoholic steatohepatitis
NAFL: Nonalcoholic fatty liver
GSEA: Gene set enrichment analysis
GO: Gene Ontology
NAS: NAFLD activity score
FDR: False Discovery Rate
FC: Fold change
STEM: Short Time-series Expression Miner
PPI: Protein–protein interaction
NES: Normalized enrichment score
