# Supplementary figures and images for: Dynamic co-expression modular network analysis in nonalcoholic fatty liver disease
Source: Hereditas. 2021 Aug 21;158:31. doi: 10.1186/s41065-021-00196-8 (PMC8380347; doi:10.1186/s41065-021-00196-8)

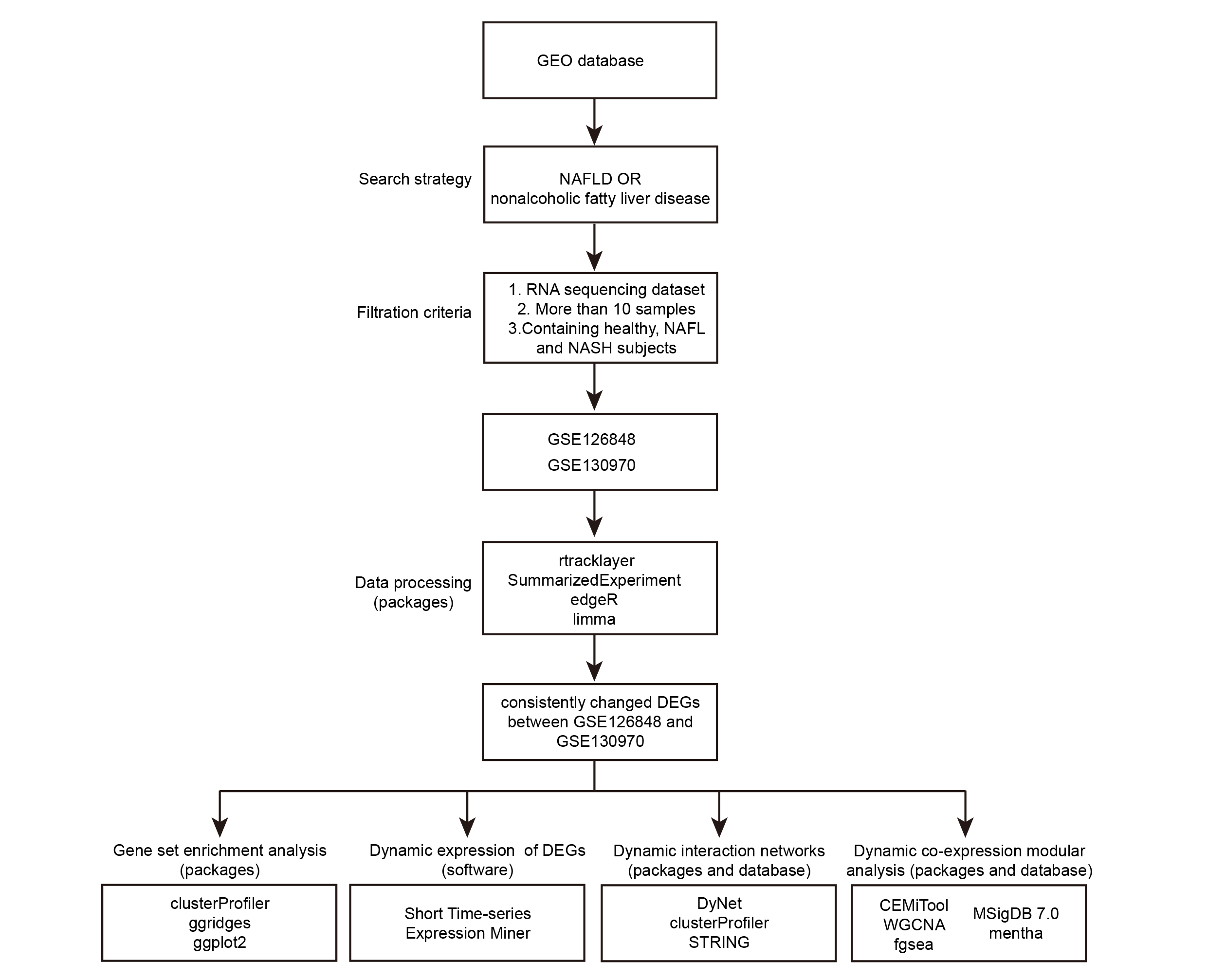

Supplement: Supplementary file 1 — Additional file 1: Supplementary figure 1. The flow diagram of dynamic network analysis. [file 41065_2021_196_MOESM1_ESM.jpg]
