# Supplementary material for: Dynamic co-expression modular network analysis in nonalcoholic fatty liver disease
Source: Hereditas. 2021 Aug 21;158:31. doi: 10.1186/s41065-021-00196-8 (PMC8380347; doi:10.1186/s41065-021-00196-8)
Supplement: Supplementary file 2 — Additional file 2: Supplementary table 1. DEGs in different profiles. [file 41065_2021_196_MOESM2_ESM.docx]

Supplementary table1. DEGs in different profiles

| Profile | DEGs |
| --- | --- |
| Profile 0 | ADCYAP1R1, ASS1, ATP6V1B1, CBLIF, CDH17, CEACAM5, CEACAM7, DIO3, DNM1, EGR1, GPR88, IGFBP2, P4HA1, PCDH20, SLC22A10 |
| Profile 1 | EFCAB12, GPR27, LUZP2, OOEP, RDH12, TNR |
| Profile 3 | ACER1, C3ORF20, FBN3, INA, MOV10L1, MUCL1, PCK1, RASL10B, RP1, SLCO1A2, SPTSSB, TFCP2L1, TMEM108, TSPAN5 |
| Profile 6 | CTCFL, DRAXIN, KRT6A, MAB21L3, MYRFL, PIWIL1, PRH2, RFX6, SLC16A4, SLC29A4, TRIM29 |
| Profile 12 | AASS, B3GAT1, CMYA5, CYP1A2, EFCAB1, GNMT, IGF1, PACSIN3, PGA3, PGA5, RIMBP2, RNF152, SH3PXD2A |
| Profile 14 | EFHD1, KRT85, LCE2D, LMX1A, UGT1A3 |
| Profile 15 | AQP5, ARHGEF4, CA6, CALML5, DCD, DSG3, EN1, FAM9B, FRMPD3, FRZB, HEPACAM, IGF2BP1, ITPRID1, KRT14, KRT15, KRT6B, KRT77, MS4A12, MYOZ2, NCS1, NSUN7, NXPE1, PIP, PNLIPRP3, REG1B, SCGB1D2, SCGB2A2, SLC27A6, TUBA3E, TYRP1, VIL1 |
| Profile 46 | CHRNB4, EME1, HDC, KCNB1, LRRC19, OLFM2, PNMT, PRKCE, RGL4, SLC5A10, SPTBN5, STRIP2, TECTB, TMEM272, TNFRSF25, ZNF385D, ZNF454 |
| Profile 47 | ACKR3, CXCL8, DCX, KCNJ5, ME1, OXT, ZMAT3 |
| Profile 49 | ACSL4, AJUBA, APOL3, ARRDC4, BAX, CAPG, CCL20, CDC6, CDKN1A, CHST9, CKS2, CTSD, DDIT4, DEFB1, DTNA, ELOVL2, F2RL1, FABP5, FAIM, FAM129B, FAT1, FBXO2, FCAMR, FGF21, FMO1, FRMD7, HBA2, IFI6, JUN, KPNA2, LGALS3, LYPD1, LYZ, MANF, NBPF15, PCLO, PDE11A, PLIN2, PRAMEF10, PRKAA2, S100A4, SDCBP2, STMN2, TAGLN2, TP53I3, TREM2, UBD |
| Profile 54 | DGKK, EDAR, EIF2S3B, GRIN2B, HSFX2, KEL, RNASE2, SMPD3, SVOP, TNFAIP6, ZNF257 |
| Profile 55 | ADAMTS4, CCL17, CYP7A1, DAZL, EEF1A2, GRID1, KCND2, LPAR3, MROH2A, NEFL, NEK10, PADI1, PCDHGA7, PSD2, TAS2R60, TMEM238L, TP53INP1, TRIM9 |
| Profile 56 | CALY, CRP, GALR3, OR2W3, VSTM5 |
| Profile 57 | AP003108.2, CCL18, CENPK, DDX47, GABBR2, GINS1, HBG1, INHBE, IP6K3, ISM1, KCNK7, MCM10, MSLN, PEG10, SERPINE1, SHC4, SLC22A13, ZNF233, ZWINT |
| Profile 58 | ALAS2, AVPR1A, CXCL11, HBG2, KCNE5, SLFNL1, TFAP2E, XK, ZDHHC2 |
| Profile 59 | AMT, BCL2L14, CENPA, CFAP99, CHIT1, COL5A3, CSPG5, CXCL10, CXCL9, CYP4F22, ENO3, FNDC5, GDPD3, HBB, HBD, HYDIN, ILDR2, ITGA6, KCNE1, KDM8, LGALS2, LHX9, NEK2, PCDHA13, PPP1R9A, PRRG3, PZP, RAPSN, RET, SCD, SGCB, SLC4A1, SNAP25, TCAP, TMEM154, TMEM45B, TPSG1, TYMP, WNT11 |
